# Use of Nutritional Supplements Based on L-Theanine and Vitamin B6 in Children with Tourette Syndrome, with Anxiety Disorders: A Pilot Study

**DOI:** 10.3390/nu14040852

**Published:** 2022-02-18

**Authors:** Renata Rizzo, Adriana Prato, Miriam Scerbo, Federica Saia, Rita Barone, Paolo Curatolo

**Affiliations:** 1 Child and Adolescent Neurology and Psychiatric Section, Department of Clinical and Experimental Medicine, Catania University, 95124 Catania, Italy; adrprato@unime.it (A.P.); mimiscerbo@gmail.com (M.S.); federicasaia@live.com (F.S.); rbarone@unict.it (R.B.); 2 Department of Cognitive Sciences, Psychology, Education and Cultural Studies, University of Messina, 98121 Messina, Italy; 3 Child Neurology and Psychiatry Unit, Systems Medicine Department, University of Rome Tor Vergata, 00133 Rome, Italy; curatolo@uniroma2.it

**Keywords:** Tourette syndrome, anxiety, psychoeducation, nutritional supplements, vitamin B6, L-theanine, nutrients

## Abstract

Background: Tourette syndrome (TS) is a neurodevelopmental disorder characterized by tics and co-occurring disorders. It has been suggested that anxiety occurs in 2–45% patients affected by Tourette syndrome. Despite dietary and nutritional factors have been found to affect a range of neurological conditions, no more studies have investigated the relationship between nutritional supplements and tics. Objective: To evaluate the effectiveness of supplementation of both L-Theanine and Vitamin B6 in reducing tics and co-occurring disorders in a sample of youth with chronic tic disorder (CTD) or Tourette syndrome with anxiety symptoms. Design: A open-label trial. Patients affected by Tourette syndrome were randomized to receive nutritional supplements based on L-Theanine and vitamin B6, or psychoeducation (PE). Participants: 34 children (30 boys and 4 girls) aged between 4 and 17 years affected by Tourette syndrome or chronic tic disorder, associated with anxiety symptoms. Results: Patients in both groups showed a reduction in the severity of tic and anxiety symptoms. Supplementation with L-Theanine and vitamin B6 was significantly more effective than psychoeducation in reducing tics and co-occurring disorders, as measured by neuropsychological findings. Conclusions: Supplementation of both L-Theanine and Vitamin B6 may help in the treatment of tic disorders associated with anxious symptoms. Between-group differences in clinician-rated severity did reach statistical significance only for tics. Despite this finding, further placebo-controlled trials are needed.

## 1. Introduction

Tourette syndrome (TS) is a highly heritable neurodevelopmental disorder characterized by multiple motor tics and one or more vocal tics that occur for more than a year, with an onset age before 18 years [1]. Most patients with TS report a range of comorbid psychopathologies that can affect patients with TS to a greater extent than tics themselves [2,3]. Co-occurring disorders of attention, impulse control and mood are common, particularly attention deficit hyperactivity disorder (ADHD) and obsessive-compulsive disorder (OCD) [4,5,6]. Other comorbidities observed in TS patients include anxiety, depression, autism spectrum disorder (ASD), learning disorders, sleep disorders [7,8,9].

The reported prevalence of comorbid anxiety in individuals with TS is variable depending on the ages evaluated and study methodologies used, ranging from 2% to 45% [3,8]. Patients affected by TS and comorbid anxiety present an earlier onset of anxiety, typically within a year onset of tics [7]; it was also reported a high-risk of onset of anxiety from four years of age [7].

No more studies have focused on the differences between anxiety in TS and anxiety in the general pediatric population. The proportion of TS patients impacted by anxiety symptoms is likely to be even greater than these estimates; patients who do not meet criteria for formal diagnosis of an anxiety disorder may nonetheless have impairing anxiety symptoms [10].

The management of TS is a challenge for clinicians, given the high interindividual variability of symptoms and the possible association with comorbid conditions that may interfere with the treatment effects for the tics. Non-pharmacologic and/or pharmacologic interventions should be considered in addition to behavioural and psychosocial interventions for TS patients with clear impairment associated with the tics, either at first referral or later, due to exacerbation of symptoms [11].

Approved pharmacological treatment consists of alpha-2-adreno-receptor-blockers, and antipsychotics. Both groups of medications are associated with mild to severe side effects including sedation, cardiovascular dysregulation, extrapyramidal motor symptoms (EPMS), sexual dysfunction, weight gain or cardiac risks that are scarcely tolerated by patients [12]. Based on guidelines, the neuropsychological intervention with the best evidence is a comprehensive intervention for tics (CBIT) [13,14]. Thus, while pharmacotherapy and behavioural treatments are effective interventions for tics, there are still a significant number of TS patients who do not tolerate or benefit from these methods of treatment. There are important limitations for behavioural approach, including age of patients, tic severity, comorbidities associated, cost, treatment availability, and insurance coverage [15].

Although dietary and nutritional factors have been found to affect a range of neurological conditions, few studies with contradictory findings have investigated the relationship between nutritional supplements and tic symptomology.

Vitamin B6 (pyroxidine) is a hydrosoluble vitamin with a wide therapeutic margin. Vitamin B6 has been shown to participate in oxidative deamination, transamination, and decarboxylation; it also participates in the decarboxylation of glutamic acid to GABA, from DOPA to dopamine and from 5-hydroxytrytophan to serotonin. It also presents anti-convulsant properties and seems to exercise a neuroprotective and detoxificant effect [16]. Vitamin B6 can be administered via food intake to children and is associated with few side effects.

L-Theanine is an amino acid primarily found in the green tea plant (*Camelia sinensis*) and some other plant extracts that are generally well tolerated. It has been associated with several health benefits, including improvements in mood, cognition and a reduction of stress and anxiety-like symptoms [17,18,19,20].

So far, no studies tried to analyse the effects of a combination of both L-Theanine and Vitamin B6 in treating TS in children also affected by anxiety symptoms. The focus of the current study was to evaluate the effectiveness of supplementation of both L-Theanine and Vitamin B6 in reducing tics and co-occurring disorders in a sample of youth with chronic tic disorder (CTD) or TS with anxiety symptoms.

## 2. Materials and Methods

### 2.1. Study Design

An open-label trial was conducted at the Child and Adolescent Neurology and Psychiatry of the Medical and Experimental Department of Catania University. Participants with a diagnosis of CTD or TS according to the Diagnostic and Statistical Manual for Mental Disorders (DSM-V), associated with anxiety symptoms, were enrolled. All patients underwent physical and neurological examination by an expert team of child and adolescent neurologists, to rule out eventually associated diseases.

Participants were randomly assigned into two groups using a simple randomization plan based on a random number list in a ratio of 1:1, the “N-group” (*n* = 17), which didn’t receive the nutritional supplement for two months, and the “THE-group” (*n* = 17), which received the nutritional supplements L-Theanine (200 mg/day) and vitamin B6 (2.8 mg/day) for two months. Based on the previous clinical trials on neurodevelopmental disorders, dose selection for L-Theanine and vitamin B6 was considered safe and well tolerated to our patients [19,21,22,23,24,25,26,27].

Psychoeducation was also conducted over eight weekly sessions in patients included in the “N-group”. Prior to enrolment, all participants provided written informed consent after receiving a complete explanation of the study and the assurance that the decision to participate in the study would not interfere with their treatment in any way. The local Ethics Committee approved this study. Enrolment began in February 2021.

### 2.2. Participants

34 children (30 boys and 4 girls) aged 4–17 years with a diagnosis of TS or CTD according to DSM-V criteria, associated with anxiety symptoms, were enrolled. The inclusion criteria were tics of moderate severity as measured by the Yale Global Tic Severity Scale (YGTSS; >13 for subjects affected by TS and >9 for those affected by CTD) [28], anxiety symptoms of reduced severity as assessed by the Multidimensional Anxiety Scale for Children (MASC; >30) [29], and an intelligence quotient (IQ) > 80.

We excluded patients older than 18 years, with an intelligence quotient (IQ) ˂ 80, who showed other primary psychiatric disorders, different from TS or CTD. The exclusion criteria also included other comorbid disorders such as autism spectrum disorder (ASD), schizophrenia spectrum disorder, conduct disorder, major depression, psychosis, or addiction. All patients were without medication during the study. During the follow-up visits parents were asked whether there had been any adverse events. Parents were also provided with a 24-h accessible phone contact for reporting any unexpected side effects requiring immediate medical intervention.

### 2.3. Clinical Assessment

The clinical assessment of the patients was performed at two time points during the study by pediatric neuropsychiatrists with solid experience in tic disorders and possible comorbidities. Participants underwent the first assessment at baseline (T0), the second after two months (T1). At T0, the Wechsler Intelligence Scale for Children (WISC-IV) was administered to evaluate the IQ of patients [30]. At baseline point (T0), patients were also assessed according to Yale Global Tic Severity Rating Scale (YGTSS), Children’s Yale-Brown Obsessive-Compulsive Scale for Children (CY-BOCS), Multidimensional Anxiety Scale for Children (MASC) and Child Depression Inventory (CDI). Considering that MASC scales are intended for children below eight years of age, in these patients we also administered the Child Behavior Checklist (CBCL), analyzing the raw score of the areas anxious/depressed to confirm the presence of anxiety symptoms [31].

After two months (T1), changes in symptoms severity were evaluated by improvement in the YGTSS and MASC scales. Those children who showed a reduction of at least 30% in YGTSS and MASC scores have been considered as “responders”.

### 2.4. Measures

The YGTSS is a clinician-rated scale used to assess the motor and phonic tic severity considering the number, frequency, duration, intensity, and complexity of tics [28]. It consists of separate motor and vocal tic checklists scored from 0 to 5 on two subscales for motor and vocal tics. The subscales were combined to produce a total tic severity score (ranging from 0 to 50). Another score ranging from 0 to 50 was assigned for global impairment due to tics [28]. The CDI has been designed to measure the self-rated assessment of depressive symptoms for children and adolescents aged between 7 to 17 years [32]. It consists of 27 items presented as three statements of varying symptom severity.

To evaluate OCD, commonly associated with TS or CTD, the CY-BOCS, a semi-structured clinician-administered interview assessing the severity of obsessions and compulsions occurring over the past week across five areas (time, interference, distressing nature, effort to resist, control over obsessions and compulsions) was also administered [33]. The CY-BOCS presents a total score ranging from 0 to 40. It is also possible to evaluate an obsession and a compulsion score separately.

Finally, all participants completed the MASC, a self-report scale that robustly represents the factor structure of anxiety in children aged 8–18 years [29]. The MASC is a standardized, 39-item measure of anxiety, through a total score and four empirically derived factor index scores: Social Anxiety, Separation Anxiety, Harm Avoidance, and Physical Symptoms. In patients below eight years of age, we also administered the Child Behavior Checklist (CBCL), analyzing the raw score of the areas anxious/depressed [31].

### 2.5. Psychoeducation

Psychoeducation provides patients and/or families with information about the features of the disorder and its etiology, comorbidities, and prognosis, and orients patients and families to the procedures and demands associated with treatment [34]. This approach which aims to reduce anxiety and emphasize the patients ‘strengths, was conducted over eight sessions. The first two sessions lasted 60 min each, then the following six sessions were 45 min each. Patients were assigned to a child/adolescent psychiatrist who was qualified CBT psychotherapist. Psychoeducation was performed with patients and parents together. In the eight sessions, patients and/or parents received information on the course, genetics, underlying neurobiology, and clinical manifestations of TS or CTD. Additionally, participants received supportive psychotherapy in which they were allowed to discuss tics and associated symptoms.

### 2.6. Composition of the Nutritional Supplements

1 mL of the nutritional supplement contains 2.8 mg of Vitamin B6 and 200 mg of L-Theanine administered in drops.

### 2.7. Statistical Analysis

Data were analyzed using SPSS software (SPSS, Inc., Chicago, IL, USA, IBM, Somers, NY, USA). Baseline demographic characteristics of participants and their clinical outcomes at baseline and 2 months after randomization were summarized by randomized group using mean (SD) for continuous data or count (%) for categorical data. Students’ t-tests were used to compare the neuropsychological scores and characteristics between groups. A *p*-value ≤ 0.05 was considered to indicate statistical significance. YGTSS and MASC outcomes among T0 and T1 were also evaluated to identify patients who showed a clinical response (responders), with a reduction at least 30% in YGTSS and MASC scores.

## 3. Results

### 3.1. Sample Characteristics

In this pilot study, we enrolled a total of 34 subjects aged 4–17 years (Mean age = 10.4 ± 3.5; male (M)/female (F) = 30:4; male = 88.2%). Of this cohort, 30 patients were affected by TS, 4 patients had a diagnosis of CTD; all patients presented also with anxiety symptoms. Patients affected by TS or CTD were randomly assigned to the “N-group” (*n* = 17) or the “THE-group” (*n* = 17), using a simple randomization plan based on a random number list. Demographic data and clinical features of all participants are displayed in Table 1. Enrolled patients were followed up by pediatric neuropsychiatrists with solid experience in tic disorders and other neurodevelopmental disorders. The use of L-theanine and Vitamin B6 in combination did not show any consistent adverse effects over the course of the trial, and no other symptoms were reported by the participants.

At baseline, no statistically significant differences were observed based on neuropsychological findings in the N-group versus the THE-group (Table 2). 

### 3.2. YGTTS Outcome

**Figure 1:**
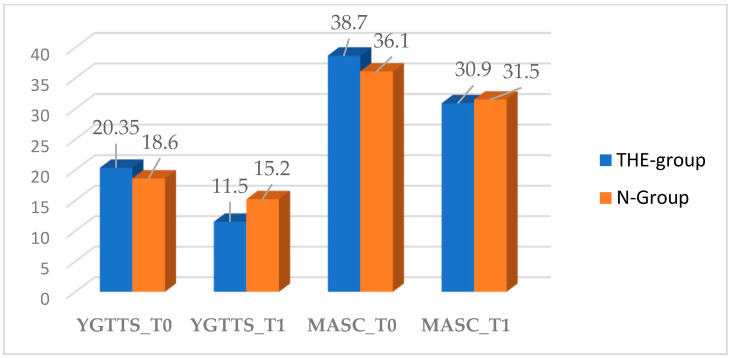
YGTSS and MASC scores between clinical groups.

In general, patients in the THE and N groups showed a reduction in the severity of tic symptoms, as assessed by YGTSS scores and sub-scores, at T1. Mean YGTSS score at 2 months after randomization was 11.5 (SD 6.1) in the THE-group compared with 15.2 (SD 4.1) in the N-group (Table 3). The mean total decrease in YGTSS at 2 months was 8.85 (43.5%) in the THE-group versus 3.4 (18.3%) in the N-group (Table 3). Statistically significant differences were observed between the THE-group versus N-group in the severity of tics as assessed by YGTSS at T1 (*p* = 0.0460). The variations in YGTSS scores are shown in Figure 1. Notably, supplementation with L-Theanine and vitamin B6 was significantly more effective than PE in reducing YGTSS scores.

**Figure 2:**
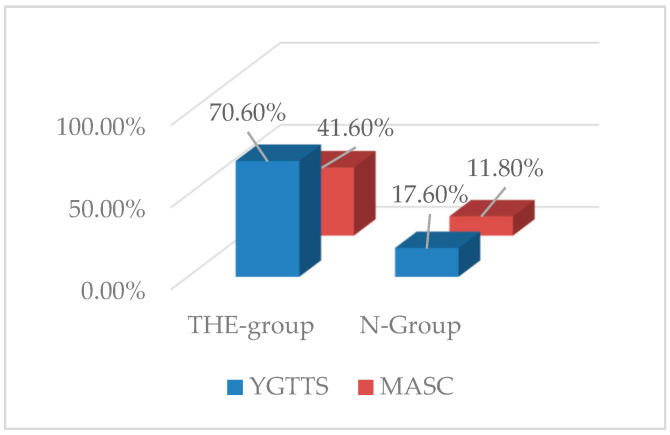
Patients who achieved a clinical response from baseline (T0) at T1.

Furthermore, 70.6% (*n* = 12) of patients in the THE-group achieved a ≥30% reduction in YGTSS scores from baseline (T0) at T1, while only 17,6% (*n* = 3) of patients of PE-group showed a similarly improvement in YGTTS scores (Figure 2).

### 3.3. MASC Outcome

Patients in the THE and N groups showed an improvement in MASC scores at T1. Mean MASC score at 2 months after randomization was 30.9 (SD 9.4) in the THE-group compared with 31.5 (SD 8.4) in the N-group (Table 3). The mean total decrease in MASC at 2 months was 7.8 (20.2%) in the THE-group versus 4.6 (12.7%) in the N-group (Table 3). No statistically significant differences were observed between the THE-group versus N-group in the severity of anxiety symptoms as assessed by MASC at T1 (*p* = 0.85). The variations in MASC scores are shown in Figure 1. In patients below 8 years of age, the presence of anxiety symptoms was confirmed administering the anxiety subscale of CBCL. In these patients, CBCL scores were higher than the reference normal values.

Furthermore, 41.6% (*n* = 7) of patients in the THE-group achieved a ≥30% reduction in MASC scores from baseline (T0) at T1, while none of the N-group showed a similarly improvement in MASC scores. Instead, 11.8% (*n* = 2) of patients of N-group presented a ≥25% reduction in MASC scores (Figure 2). Moreover, the effect of nutritional supplementation on anxiety symptoms was higher in comparison with psychoeducational treatment.

## 4. Discussion

This pilot study investigates the efficacy of L-Theanine and Vitamin B6 supplementation compared with PE in reducing tics and anxiety symptoms in youths with TS or CTD with comorbid anxiety disorder. The results of this trial show that both interventions contribute to decrease of tics and anxiety symptoms as measured by the YGTSS and the MASC. Furthermore, supplementation with L-theanine and vitamin B6 is more effective in reducing tic and anxiety symptoms, compared to psychoeducational treatment. However, the difference between both groups resulted significative only in the case of YGTSS scores. As far as we know, the comorbidity of TS and anxiety disorders has not been intensively studied. Vermilion et al. [35] recently found that youth with tic disorders have significantly greater anxiety severity compared to youth from the community, and similar total anxiety symptom severity compared to treatment-seeking anxious youth. Therefore, anxiety symptom phenotype may differ in youth with and without tic disorders, which may have implications for targeting anxiety treatment in youth with tic disorders [35]. In fact, the distress and burden associated with the co-existing problems, such as mood disorders or anxiety, is often more significant to TS patients than the tics themselves. Although the presence of a comorbid condition may have important implications for the choice of medication, there are no treatment studies to guide the clinician in treating these co-existing problems [11]. A few data from the literature have investigated the efficacy of nutritional supplements in the treatment of tic disorders and other neuropsychiatric conditions in pediatric cohorts. Garcia-Lopez et al. [16,25] investigated the effectiveness and safety of magnesium and vitamin B6 with respect to placebo treatment and demonstrated that a collateral treatment with magnesium and vitamin B6 could improve control of the illness and help reduce side effects. The treatment assayed was safe and effective in alleviating the harmful effects of TS in children, by a reduction of total tics score measured by YGTSS [25]. Mantel et al. [36] also described benefits in the reduction of tics after the incorporation of nutritional and dietary changes [36]. Another recent study conducted on children with TS compared with typically developing (TD) children demonstrated a clinical improvement in 75% of supplement users in the TS group, mainly in motor and vocal tics, sleep quality and anxiety reduction [37]. Other studies have reported the beneficial effects of vitamin B6 and nutritional supplements in the treatment of ASD [26,27,38]. Furthermore, vitamin B6 eventually associated with magnesium supplementation, could provide a meaningful clinical benefit in daily life for adult’s individuals with stress [39,40]. Hannant et al. [41] also explored the effects of GABA Oolong (GABA 279 mg/100 g, L-Theanine 104.48 mg/100 g) in children with ASD and demonstrated an improvement in manual dexterity and sensory responsivity. Lyon et al. [22] have also demonstrated that L-theanine is safe and effective in improving some aspects of sleep quality in boys diagnosed with ADHD [22]. Moreover, published data regarding adult patients suggests that L-theanine administered at daily doses ranging from 200 to 400 mg for up to 8 weeks are safe and induce anxiolytic and anti-stress effects in acute and chronic conditions [17,18,19,42]. Another randomized, placebo-controlled study conducted on patients with schizophrenia and schizoaffective disorder had speculated that L-theanine augmentation of antipsychotic therapy could improve their positive, activation and anxiety symptoms [23]. Recently, a 20-week open label proof-of-concept study was undertaken involving 28 participants with pharmacological-resistant OCD treated with a nutraceutical combination: N-acetyl cysteine, L-theanine, zinc, magnesium, pyridoxal-50 phosphate, and selenium. Statistically significant improvements were revealed on the YBOCS total score and secondary outcomes, suggesting the potential usefulness of this treatment option especially for patients with lower symptom levels [24].

In contrast, Sarris et al. [21] conducted a 10-week study involving participants with a diagnosis of generalized anxiety disorder (GAD); their results did not support the efficacy of L-theanine in the treatment of anxiety symptoms in GAD [21]. However, participants of this double-blind, randomized trial received adjunctive L-theanine or matching placebo with their current stable antidepressant treatment and/or concurrent psychotherapy; in this case, L-Theanine treated participants could potentially be more challenging to treat (due to non-response to both antidepressant and psychological therapy, and a higher mean age) [21].

Despite the lack of a clear evidence base to support the idea that changes in diet could alleviate some of the symptoms of TS, future studies on dietary and nutritional factors in TS may be useful to improve our understanding of possible alternative interventions [43]. To the best of our knowledge, this is the first study to highlight the efficacy of a combination of both L-Theanine and Vitamin B6 in reducing the severity of tic and anxiety symptoms. It is worth noting that the results from our study highlighted that both interventions are effective in improving tics and anxiety symptoms, but only the effect on tics is statistically significative for patients of THE-group. Significantly greater reductions in tics as assessed by YGTSS at T1 were found in the THE-group relative to N-group. Participants receiving supplementation with L-Theanine and vitamin B6 demonstrated a mean reduction in YGTSS score of 8.85 (43.5%), higher to that observed in the N-group (Table 3). Between-group differences in clinician-rated severity of tics did reach also statistical significance (*p* = 0.0460).

Conversely, no statistically significant differences were observed between THE-group versus N-group in the improvement of anxiety symptoms, as assessed by MASC scores. This may be probably attributable to the lower anxiety symptoms levels that we considered as inclusion criteria. It is possible to hypothesize that complementary supplementation with vitamin B6 and L-theanine may be more effective in children with higher anxiety symptoms levels.

There are several limitations in our study. First, it was an open-label design, and we cannot exclude the influence of the placebo effect on our results. Second, the study was conducted on a small sample size, and the number of participants (*n* = 34) may have been insufficient. Due to the relatively small size and, therefore, the limited power of the study, the results should be considered as preliminary rather than conclusive. Third, our study did not include a control group, and had a short follow-up period, and so a longer interventional period than 2 months may have been required to highlight the potential benefits. Finally, the difference at baseline in mean scores for YGTSS and MASC between the two groups may have an impact on outcome measures. Furthermore, the psychotropic effects of L-theanine and vitamin B6 may be more significant in several clinical cases. More studies are also warranted to confirm the beneficial effects in a much larger sample size. Further trials comparing the combination of vitamin B6 and L-Theanine with the two components independently are also necessary.

On the other hand, the strengths of the study include its randomized and controlled design, and thoroughly considered and implemented inclusion and exclusion criteria. The possibility of a complementary treatment with vitamin B6 and L-theanine would represent an important improvement in controlling the illness, by reducing the need for neuroleptic drugs and other medication; it would also reduce the amount and severity of side effects. L-Theanine and Vitamin B6 present very few side effects and have a long history of therapeutic use.

In conclusion, our findings suggest that supplementation of both L-theanine and vitamin B6 may assist in the reduction of tics and anxiety in children affected by TS or CTD with comorbid anxiety symptoms.

## 5. Conclusions

This study highlights that complementary supplementation with vitamin B6 and L-theanine can help in the treatment of tic disorders associated with anxious symptoms. Effects on tic symptoms and anxiety levels are more evident in THE-group, which received the nutritional supplement. Moreover, between-group differences in clinician-rated severity did reach statistical significance only for tics.

Despite this finding, further placebo-controlled trials are needed to improve our knowledge about the effects of this nutritional supplement in treating patients with TS or CTD also affected by anxious symptoms.

## Figures and Tables

**Table 1:** Demographic and clinical features of the participants.

Partecipant Characteristics	Total Sample (*n* = 34)	THE-Group (*n* = 17)	N-Group (*n* = 17)	*p*-Value
Male (%)	30 (88.2%)	15 (88.2%)	15 (88.2%)	1
Mean age (years) ± SD	10.4 (±3.5)	9.3 (±3.9)	11.5 (±2.7)	0.6078
Tic disorders	4 (11.8%)	3 (17.6%)	1 (5.9%)	0.157
TS	30 (88.2%)	14 (82.4%)	16 (94.1%)	0.287

**Table 2:** Neuropsychological findings of the TS patients at baseline (T0).

Measures	Total Sample (*n* = 34)	THE-Group (*n* = 17)	N-Group (*n* = 17)	*p*-Value
TIQ	97.5 (±9.9)	99.7 (±12.1)	96.5 (±7.6)	0.3627
YBOCS	3.8 (±5.9)	3.2 (±5.7)	4.5 (±6.2)	0.5290
CDI	2.9 (±4.5)	2.9 (±4.96)	2.9 (±4.1)	1
YGTSS	19.5 (±5.6)	20.35 (±5.8)	18.6 (±5.4)	0.3694
MASC	37.4 (±8.5)	38.7 (±8.8)	36.1 (±8.35)	0.3835

**Table 3:** Comparisons between mean YGTTS and MASC values at T1.

	THE-Group (*n* = 17)	N-N-Group (*n* = 17)	*p*-Value
YGTSS			0.0460
Mean values	11.5 (±6.1)	15.2 (±4.1)	
Mean total decrease	8.85 (43.5%)	3.4 (18.3%)	
MASC			0.8457
Mean values	30.9 (±9.4)	31.5 (±8.4)	
Mean total decrease	7.8 (20.2%)	4.6 (12.7%)	

## Data Availability

The data presented in this study are available on request from the corresponding author.
